# The Complete Mitogenome of *Elymus sibiricus* and Insights Into Its Evolutionary Pattern Based on Simple Repeat Sequences of Seed Plant Mitogenomes

**DOI:** 10.3389/fpls.2021.802321

**Published:** 2022-01-26

**Authors:** Yanli Xiong, Qingqing Yu, Yi Xiong, Junming Zhao, Xiong Lei, Lin Liu, Wei Liu, Yan Peng, Jianbo Zhang, Daxu Li, Shiqie Bai, Xiao Ma

**Affiliations:** ^1^College of Grassland Science and Technology, Sichuan Agricultural University, Chengdu, China; ^2^Sichuan Academy of Grassland Science, Chengdu, China

**Keywords:** *Elymus sibiricus*, seed plant, HGT, SSR, mitochondrial genome

## Abstract

The most intriguing characteristics of plant mitochondrial genomes (mitogenomes) include their high variation in both sequence and structure, the extensive horizontal gene transfer (HGT), and the important role they play in hypoxic adaptation. However, the investigation of the mechanisms of hypoxic adaptation and HGT in plant mitochondria remains challenging due to the limited number of sequenced mitogenomes and non-coding nature of the transferred DNA. In this study, the mitogenome of *Elymus sibiricus* (Gramineae, Triticeae), a perennial grass species native to the Qinghai-Tibet plateau (QTP), was *de novo* assembled and compared with the mitogenomes of eight Gramineae species. The unique haplotype composition and higher TE content compared to three other Triticeae species may be attributed to the long-term high-altitude plateau adaptability of *E. sibiricus*. We aimed to discover the connection between mitogenome simple sequence repeats (SSRs) (mt-SSRs) and HGT. Therefore, we predicted and annotated the mt-SSRs of *E. sibiricus* along with the sequencing of 87 seed plants. The clustering result based on all of the predicted compound mitogenome SSRs (mt-c-SSRs) revealed an expected synteny within systematic taxa and also inter-taxa. The mt-c-SSRs were annotated to 11 genes, among which “(ATA)3agtcaagtcaag (AAT)3” occurred in the *nad5* gene of 8 species. The above-mentioned results further confirmed the HGT of mitogenomes sequences even among distant species from the aspect of mt-c-SSRs. Two genes, *nad4* and *nad7*, possessed a vast number of SSRs in their intron regions across the seed plant mitogenomes. Furthermore, five pairs of SSRs developed from the mitogenome of *E. sibiricus* could be considered as potential markers to distinguish between the species *E. sibiricus* and its related sympatric species *E. nutans*.

## Introduction

As the largest of all known organelle genomes, the plant mitochondrial genome exhibits intriguing features including the lowest known rates of synonymous substitution, relatively abundant rearrangement, and high level of inversion and recombination. Meanwhile, plant mitochondria are the only organelles with a propensity to integrate foreign sequences *via* intracellular and horizontal gene transfer (HGT) (Vaughn et al., 1995). A large number of mitochondrial genes in higher plants were horizontally transferred early in the process of evolution, though this process has virtually ceased among animals and fungi (Maréchal and Brisson, 2010). Most of the transferred mitochondrial genes are ribosomal protein encoding genes, along with the infrequent transfer of respiratory genes (Adams and Palmer, 2003). The transferred genetic information is not only limited to complete functional genes, but also includes some non-coding sequences and gene segments (Adams and Palmer, 2003). HGT improves the genetic diversity of species through the substitution of lost genes or the formation of chimeric genes (Hao and Palmer, 2011). However, the usual non-coding nature of the transferred DNA and the limited complete genome sequences of mitochondria have restricted the exploration of the explicit evidence for HGT. Simple sequence repeats [SSRs, i.e., microsatellites in the typical form of “(repeats motif)_n_”] are useful to perform genetic and evolutionary studies due to their abundant alleles per locus and wide distribution in the genome (Qin et al., 2015). The flanking sequences of a specific SSR in the genome are usually conserved, which lead to the similar feature of cross-species transfer as mitochondrial DNA. This is expected to provide new evidence for the HGT of mitochondrial sequences from the perspective of mitochondrial SSRs (mt-SSRs). Compound SSRs, namely, c-SSRs [in the typical form of “(repeats motif)_n_-species specific sequence-(repeats motif)_n_]” range from less than 10 bp to more than 200 bp. Therefore, the contained species-specific sequences accounting for the smaller proportions of cross-species transferability of c-SSRs were always overlooked when performing genetic diversity analysis. Mitochondrial c-SSRs (mt-c-SSRs) possess both a lower rate of cross-species transferability specific to c-SSR and the dramatic variation specific to mitochondria, and it seems impossible to find the intercommunity of mt-c-SSRs sequences between two closely related species. In this context, it may be worth exploring the sequence correlations of mt-c-SSRs among species at a broader taxonomic scale, which may provide a new perspective and evidence for the HGT of plant mitochondrial DNA.

As a product of biological evolution, repeated sequences are often used to reveal the historical imprint left by the exchange and recombination of genetic material between species over long periods of time (Charlesworth et al., 1986). Eukaryotes are considered to have evolved far more repetitive proteins than prokaryotes to perform functions specific to their unique physiological needs (Marcotte et al., 1999). Only a few studies, however, have connected SSRs with their specific function in stress tolerance. One of the typical examples is microsatellite DNA isolated from the genome of the heat-tolerant *Phaseolus vulgaris* genotype, thus providing polymorphic genetic markers for heat-tolerant quantitative trait locus (QTL) linkage (Guo et al., 2000). The driving force of natural evolution and artificial selective breeding is generated by the mutation, recombination, and regulation of genes, among which regulation may play a leading role. One of the main factors controlling the recombination and regulation of structural genes are repetitive sequences (Wang et al., 2019). However, little is known about the function of such repetitive sequences and their biological significance, and most of them are only recognized as effective molecular markers. Especially for mitochondria whose gene functions have been well-investigated, the significance of SSRs and their effect on mitochondrial gene function is worthy of further exploration.

The most widely known function of mitochondria is converting the proton concentration gradient to ATP for biological activity through oxidative phosphorylation (Stechmann et al., 2008). In particular, mitochondria play a key role in the internal regulation of organisms under hypoxic conditions, which affects their function mainly through three aspects: mitochondrial oxygen consumption, mitochondrial metabolic pattern, and mitochondrial DNA copy number. On the other hand, the hypoxic acclimatization, that is, the high-altitude adaption of organisms, has been proved to correlate with the haplotype of several mitochondrial protein-coding genes like *ATP6* and *Cytb* in Tibetan goats (Gu et al., 2009). Furthermore, *Cyt b*, *COX*, and *ND* genes in mitochondria were shown to be under selection and contribute to the high-altitude adaptation of plateau pikas and Tibetan (Yang et al., 2006; Gu et al., 2012). However, no evidence has indicated the role of mitochondrial DNA in the high-altitude adaptation of plants. The main limitation is the difficulty of obtaining and assembling intact mitochondrial genome sequences from plants due to the enriched repeat sequences. Therefore, representative mitochondrial genomes such as those of native plants from the Qing-Tibetan Plateau (QTP) can act as the best resources for scrutinizing the important adaptation mechanisms of higher plants. As a widespread wild species belonging to Triticeae, Gramineae, *Elymus sibiricus* is one of the native grasses in the QTP and cold temperate zone of Asia, which possesses a strong ecological adaptability to alpine climate. The genome composition of *E. sibiricus* is StStHH, in which the maternal donor of St is derived from *Pseudoroegneria*, consistently with a congeneric allohexaploid such as *E. nutans* (with the genome composition of StStHHYY) (Yan et al., 2011). Based on the ecological niche model established using the MaxEnt method, potential evidence for the distribution of *E. sibiricus* in the QTP has been found in the Last Glacial Period or even earlier (Han et al., 2019), which makes this area as an ideal resource to study the adaptation of plants to high-altitude regions.

*E. sibiricus* cv “Chuancao No. 2” was originally bred from wild accessions in the QTP and has become the dominant forage variety with the largest populated area and the most remarkable effect of industrialization in the region. In the present study, the mitochondrial genome of *E. sibiricus* cv “Chuancao No. 2” was sequenced and assembled. Moreover, a comparative mitochondrial genome analysis was performed among eight Gramineae mitochondrial genomes and *E. sibiricus*. Five pairs of polymorphic mt-SSRs were used to construct the genetic structure of 60 wild *E. sibiricus* and 32 *E. nutans* wild accessions. Finally, benefiting from the available information of mitochondrial genomes in the National Center for Biotechnology Information (NCBI), the mitochondrial genome sequences were downloaded and their corresponding mt-c-SSRs sequences were extracted to study the connection among the 88 seed plants for the first time, which could provide valuable information of their characteristics obtained through cross-species transfer.

## Materials and Methods

### Mitochondrial DNA Extraction, *de novo* Sequencing, and Mitochondrial Assembly of *Elymus sibiricus*

The seeds of *E. sibiricus* cv “Chuancao No.2” were kindly provided by the Sichuan Academy of Grassland Science (Chengdu, China), which were cultivated in a growth chamber (25°C, 300 μmols⋅m^2^⋅s^–1^, 18 h light/6 h dark cycle). The genomic DNA was isolated from 7-day-old leaves using a DNA isolation Kit (BioVision) and the quality and concentration were detected by 1% agarose gel electrophoresis and NanoDrop 2000 (Thermo Fisher Scientific, Waltham, Massachusetts, United States). To obtain the full-length mitochondrial genome of *E. sibiricus* with high accuracy, high-throughput sequencing (Illumina) and long-read sequencing (Oxford Nanopore) were combined in this study. First, total DNA was sequenced using the Illumina Miseq platform (Illumina, San Diego, California, United States) with an average insert length of 150 bp. Thereafter, fastp (Pearson, 1990) (version 0.20.0)^1^ software was used to filter the low-quality sequences from the raw data according to the following criteria: (1) Discard the adapter and primer sequences; (2) filter the reads with average-quality values lower than Q20 (sequence accuracy higher than 99%); and (3) filtering the reads whose N (bases other than A/T/C/G) content is more than 5. The long-read sequencing technology was critical in achieving the contiguity and completeness of mitogenomes, which was then corrected with Illumina sequencing using LoRDEC (Leena and Eric, 2014). The corrected Nanopore sequencing data were assembled into contigs using canu software (Koren et al., 2017) with settings of (1) genome size of 5 Mb; and (2) correctedErrorRate = 0.03. The obtained contigs were then blasted with the plant mitochondrial gene database by BLAST v2.6.^2^ The blasted contigs were considered as the seed sequences, and were extended and cyclized referring to the raw data to obtain the circular dominant structure. Specially, the sequencing data were blasted back to the longest contig (contig A, 115,095 bp) using minimap2 (v. 2.15-r905) software (Li, 2018). The sequences with a larger than 90% similarity with contig A and overlap length larger than 3 kb were screened to extend the contig A. Repeating the extension step until the extended sequence can be connected with the beginning of contig A. This indicated that a circle molecule was obtained. The assembly result obtained above was corrected with the full-length data using NextPolish1.3.1 (Hu et al., 2019)^3^ and next-generation sequencing data using pilon software to obtain the final assembly result.

### Annotation of Mitochondrial DNA

The annotation of the *E. sibiricus* mitogenomes was performed as previously reported (Shu-Miaw et al., 2008; Guo et al., 2016). In short, the non-redundant database and the organelle genomic website of NCBI were used to annotate the protein-coding/rRNA genes and the boundaries of intron/exon, respectively. The online website open reading frame (ORF)-finder^4^ with the standard genetic code was used for identification of genes with hypothetical proteins. The tRNAscanSE (Lowe and Eddy, 1997)^5^ software was employed to annotate the tRNA genes. The circular mitogenome map was visualized using OGDRAW (Greiner et al., 2019)^6^.

### RNA Editing Prediction and Transposon Element Identification

The forward RNA editing (C to U) prediction of 11 shared genes in Gramineae was carried out through the online website of PREPACT (Henning et al., 2018).^7^ Using default parameters, the transposable element (TE) of mitochondrial genomes were identified through the CENSOR (Huda and Jordan, 2009).

### Homologous Sequence Analysis and Chloroplast-Derived Mitochondrial Sequences of Gramineae

To date, only eight mitogenomes of Gramineae species have been sequenced, including *Aegilops speltoides* (NC_022666.1), *Eleusine indica* (NC_040989.1), *Oryza sativa* (NC_011033.1), *Saccharum officinarum* (NC_031164.1), *Sorghum bicolor* (NC_008360.1), *Triticum aestivum* (NC_036024.1), *T. timopheevii* (NC_022714.1), and *Zea perennis* (NC_008331.1). To investigate the phylogenetic relationships of Gramineae species based on mitochondrial sequences, the mitogenomes of *E. sibiricus* and the eight aforementioned Gramineae species were comparatively analyzed. The homologous blocks between the mitochondrial and chloroplast (chl) genomes of these nine Gramineae species were identified and visualized using Mauve software (Darling et al., 2004). To detect the chloroplast-derived mitogenome sequences, a stricter criterion was applied using BLAST software (Cha and Rouchka, 2005) with a similarity value of 70% and *E*-value of 10E-5.

### Phylogenetic Tree Construction

The whole mitogenome sequences of the nine Gramineae species were aligned using MAFFT software (Standley, 2013) with a bootstrap value of 1,000. The maximum likelihood phylogenetic tree was visualized in Figtree software (Hampl et al., 2001).

### Prediction of Simple Sequence Repeats Prediction of *Elymus sibiricus* and Angiosperm Mitogenomes

The SSRs of *E. sibiricus* and further 87 seed plant mitogenomes were identified *via* MISA (Beier et al., 2017) using the default parameters. The annotation of predicted SSRs was performed with Perl64 with the scripts of “perl get_misa_anno.pl misa file gb file > result.xls.”^8^ Fifty pairs of the trinucleotide repetition SSR primers located in the genic regions were selected to amplify the six wild accessions of *E. sibiricus*. Five pairs of polymorphic SSR markers were finally selected for the further amplification of the 60 wild *E. sibiricus* and related 36 *E. nutans* accessions. According to the protocol of Xiong et al. (2019) with minor modifications, the SSR-PCR amplifications were carried out in a total of 10 μL reaction volume including 2 μL (20 ng⋅μL^–1^) template DNA, 0.4 μL (5 pmol⋅μL^–1^) forward and reverse primers, 5 μL mix (containing 10 × PCR buffer, Mg^2+^, and dNTPs), 0.2 μL Taq enzyme (2.5 U⋅μL^–1^), and 2 μL ddH_2_O. The following PCR program was used: predenaturation step at 94°C for 5 min, followed by 30 cycles of 30 s at 94°C, annealing for 30 s at 51–66°C, then 1 min at 72°C, and final extension for 10 min at 72°C. The amplified products were detected on 8% non-denaturing polyacrylamide gel. The primers that amplified two bands were considered as heterozygous, while those that amplified only one band were considered as homozygous. However, one pair of primers amplified three bands, probably because they were also amplified on the nuclear genome. Considering the difficulty to obtain allele dosage in allotetraploids, the amplified bands were recorded as 1/0 (presence of band/absence of band) for further study. The binary matrix was used to determine the genetic background and population structure by STRUCTURE software (Pritchard et al., 2000). Then, the compound SSRs (cSSRs) of all 88 seed plant mitogenomes were extracted and marked as “species name_1,” “species name_2,” etc. All cSSRs sequences of the 88 mitogenomes were grouped into a data set and loaded into the CD-HIT online website (Huang et al., 2010).^9^ CD-HIT-EST plate was used to cluster the above-mentioned data set with the sequence identity cutoff value of 0.9 and the default settings of other parameters. The cSSR sequences of two species with the same length and sequence identity of 100% were considered as “transferred sequences.” Considering the cross-species transferability characteristic of SSR, which depended on the repeat motif and numbers, the repeat number of mono-, di-, and tri- nucleotides of each species were counted and presented as a column of data. Thereafter, that data column was used to calculate the Spearman’s correction coefficients among the 88 species, which were then presented in a heatmap.

## Results

### Mitochondrial Genome Sequencing, Assembly, and Annotation

**FIGURE 1:**
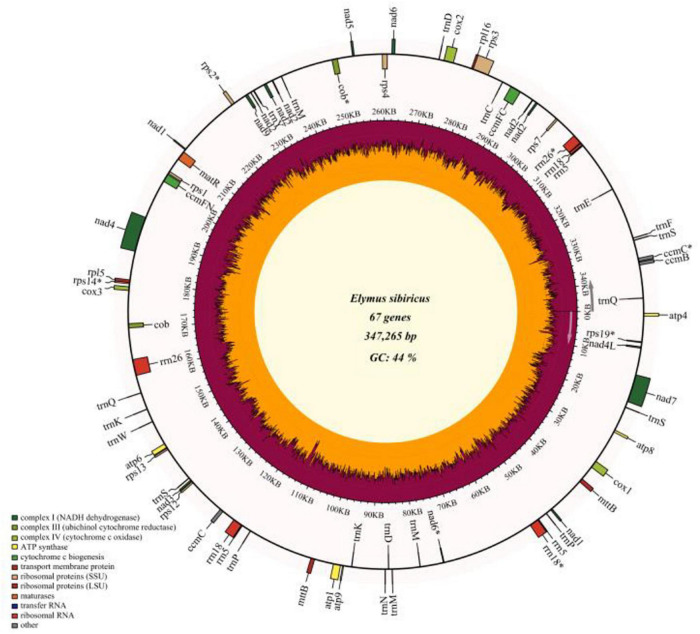
Gene map of the complete mitochondrial genome of *Elymus sibiricus*. Genes inside the outer circle are transcribed in a clockwise direction, otherwise, in a counterclockwise direction. Genes marked with an asterisk are pseudogenes. The inner circle represents guanine and cytosine (GC) content (%).

The read depths of Illumina Miseq and Single Molecular Nanopore DNA sequencing were 77.6 X and 118.7 X, and a total of 485,924 reads were obtained using Nanopore sequencing with a mean read length of 15,969 bp. The filtered reads were *de novo* assembled into contigs. A total of eight contigs (> 10 Kb, with the longest contig of 115,095 bp) were used to assemble the whole mitogenome of *E. sibiricus* with an N50 value of 46,805 bp. Finally, the mitochondrial genome of *E. sibiricus* was assembled into a single circular molecule with the size of 347,265 bp (Figure 1 and Supplementary Figure 1) and guanine and cytosine (GC) content of 44.47%. This mitochondrial genome contained 67 genes including 20 tRNAs, six rRNAs, 33 protein-coding genes, and eight pseudogenes (*rps19*, *nad6*, *cob*, *rps14*, *rps2*, *rrn26*, *rrn18*, and *ccmC*).

### Comparison Analysis of Gramineae Mitogenomes

**FIGURE 2:**
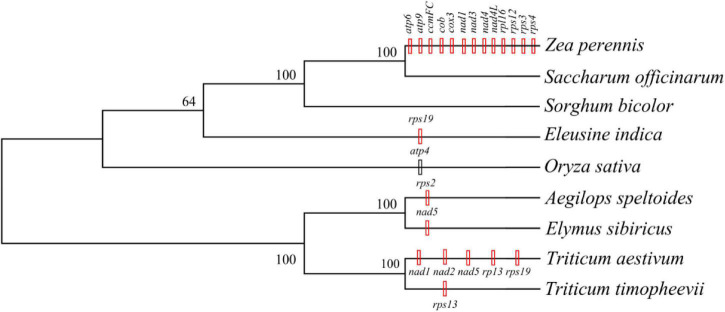
The phylogenetic tree was constructed based on the single nucleotide polymorphisms (SNPs) and indels extracted from the 11 shared genes of Gramineae species with a bootstrap value of 1,000 using the Tamura-Nei model. The black vertical line indicates gene deletion, while the red vertical line represents gene acquisition in the same or an inferior cluster.

The mitogenomes of *E. sibiricus* and another eight sequenced Gramineae species were compared in terms of gene, GC, and TE contents (Table 1). The GC content was relatively conserved but highly variable gene and pseudogene numbers were detected among Gramineae mitogenomes. Especially, the *E. sibiricus* mitogenome contained the maximum number of pseudogenes (8). The mitogenome size of *E. sibiricus* (347,265 bp) was only larger than that of *Saccharum officinarum* (300,784 bp), while it was smaller than those of the other seven Gramineae species. There was one gene acquired (*nad5*) and one gene lost (*rps2*) in the mitogenomes of *E. sibiricus* compared with *Aegilops speltoides* (Figure 2). Compared with four mitogenomes, including *Zea perennis*, *Saccharum officinarum*, *Sorghum bicolor*, and *Eleusine indica*, the mitogenome of *Oryza sativa* lost the *atp4* gene (Figure 2).

**TABLE 1:** General information of Gramineae mitogenomes.

	*Saccharum officinarum*	*Sorghum bicolor*	*Zea perennis*	*Eleusine indica*	*Oryza sativa*	*Elymus sibiricus*	*Triticum aestivum*	*Aegilops speltoides*	*Triticum timopheevii*
Accession no.	NC_031164	NC_008360	NC_008331	NC_040989	NC_011033	CNA0036159	NC_036024	NC_022666	NC_022714
Size (bp)	300,784	468,628	570,354	520,691	490,520	347,265	452,526	476,091	443,419
GC%	43.95	43.73	43.88	43.3	43.85	44.47	44.35	44.43	44.35
GC1	43.75	43.76	43.76	43.21	44.01	44.44	44.64	44.54	44.35
GC2	44.04	43.64	44.09	43.41	43.84	44.3	44.46	44.64	44.33
GC3	44.04	43.78	43.81	43.27	43.71	44.68	43.96	44.09	44.37
Genes	43	54	59	66	81	67	67	77	77
tRNAs	16	18	17	24	22	20	22	25	23
rRNAs	6	3	3	6	3	6	9	10	8
Protein coding genes	21	32	32	34	50	33	36	39	39
Pseudogene	0	1	7	2	6	8	0	3	7
TE content%	3.76	1.49	1.26	4.63	5.46	1.94	0.98	0.94	1.01

**FIGURE 3:**
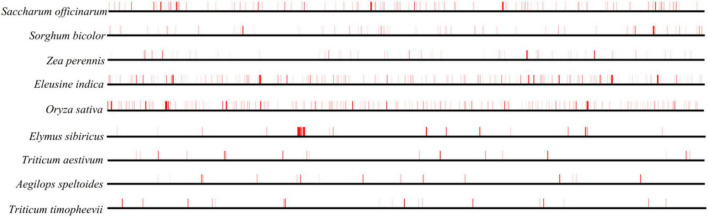
TE prediction of nine Gramineae mitogenomes. The horizontal lines in bold black represent nine mitogenomes, and the vertical red lines indicate the location of the predicted TEs.

The TEs in the nine Gramineae mitogenomes were predicted and illustrated in Figure 3, which shows their generally scattered distribution in the four Triticeae species mitogenomes and their dense distribution in other mitogenomes. The TE distribution was relatively centralized in the *E. sibiricus* mitogenome from 110,563 to 114,976 bp, corresponding to the genic region between *mttB* and *trnP*. The TE content presented great variation in the nine Gramineae mitogenomes, ranging from 0.94% (*Aegilops speltoides*) to 5.46% (*Oryza sativa*) (Table 1). In general, as opposed to *E. sibiricus*, three species of Triticeae (*Aegilops speltoides*, *Triticum timopheevii*, and *Triticum aestivum*) possessed a lower TE content compared with other Gramineae species.

**FIGURE 4:**
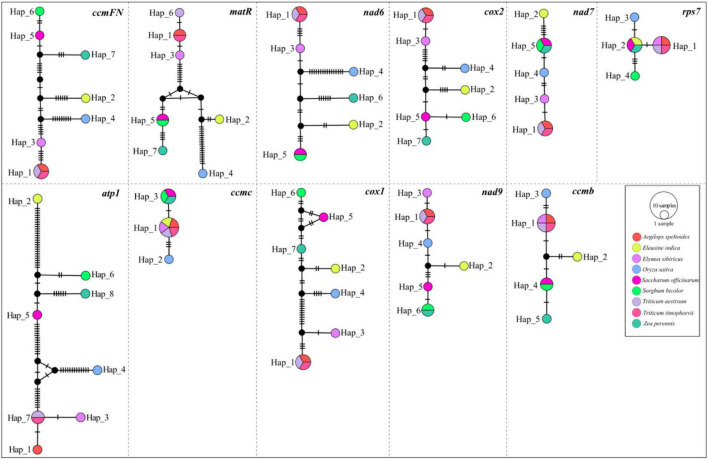
Haplotype networks constructed using 11 shared genes between Gramineae mitogenomes.

A total of 11 genes, including *atp1*, *ccmB*, *ccmC*, *ccmFN*, *cox1*, *cox2*, *matR*, *nad6*, *nad7*, *nad9*, and *rps7*, were shared among the nine mitogenomes listed in Table 1. The haplotype networks constructed based on the BLASTed sequences of these 11 genes showed high variation among the nine Gramineae species (Figure 4). It is worth noting that *Triticum timopheevii* and *T. aestivum* shared the same haplotype composition in all genes. Furthermore, all the 11 genes of *O. sativa* possessed a unique haplotype composition. The haplotypes of *E. sibiricus* genes *rps7*, *ccmC*, and *ccmB* were shared with those of the genes of Triticum species. For other species, the haplotypes of *E. sibiricus* were unique.

**FIGURE 5:**
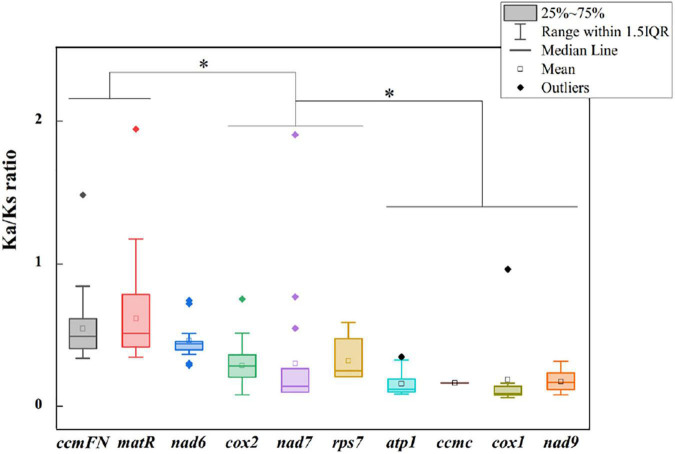
Ka/Ks ratio calculated based on 10 shared genes among the nine Gramineae mitogenomes. * indicates *P* < 0.01.

The non-synonymous/synonymous mutation ratio (Ka/Ks) between any two species was also calculated based on the 11 shared genes. According to Figure 5, the mean values of pairwise Ka/Ks of *ccmFN* and *matR* were significantly higher than those of other genes (*P* < 0.05). Further analysis showed that the pairwise Ka/Ks values were almost less than one except for *ccmFN* and *nad7* between *O. sativa* and *Eleusine indica*, and *matR* between *Zea perennis* and *Eleusine indica* or *E. sibiricus* (Supplementary Table 1).

The RNA editing sites of ten shared mitogenome genes were predicted (Supplementary Figure 2), except for *rps7*, where no RNA editing site was predicted. All of the selected Gramineae species shared the same RNA editing sites within *atp1*, *ccmC*, and *cox1*. Notably, four Triticeae species (*E. sibiricus*, *Aegilops speltoides*, *T. timopheevii*, and *T. aestivum*) possessed the same RNA editing sites in all ten genes except for *matR* and *nad6*.

### Gene Transfer Between Mito- and Chl- Genomes of Gramineae Species

Locally collinear blocks (LCBs) between mitochondrial and chloroplast genomes of the nine Gramineae species were analyzed, which showed a scattered distribution of synteny regions in the whole of mitogenomes (Supplementary Figure 3). The *18s rRNA* of all studied mitogenomes were homologous with specific regions of chloroplast genomes (Supplementary Table 2). Furthermore, *trnW*, *trnP*, *trnN*, and *trnF* in eight of the nine chloroplast genomes were homologous with specific regions of mitogenomes. In all four *Triticeae* mitogenomes, there were six genes (*rrn18*, *rrn26*, *trnF*, *trnN*, *trnS*, and *trnW*) homologous with their own corresponding chloroplast genomes.

**FIGURE 6:**
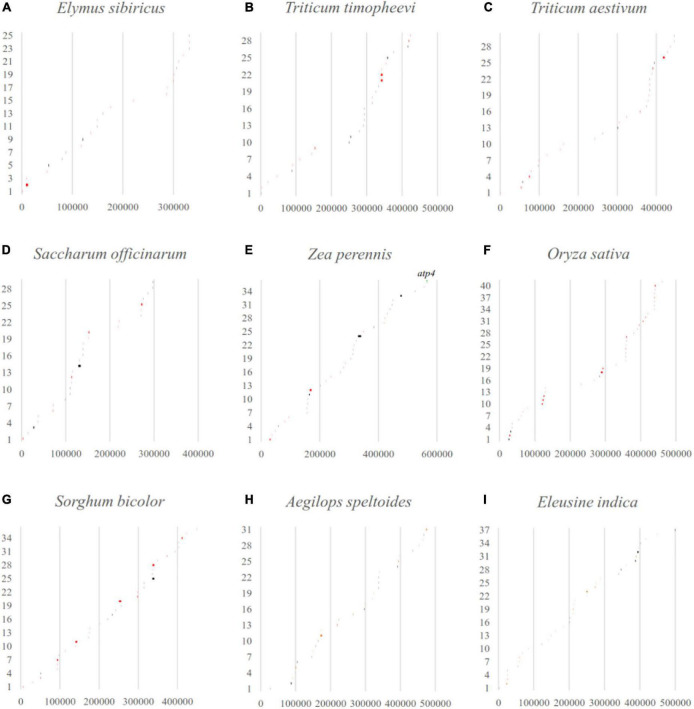
The gene transfer between mitochondrial and chloroplast genomes in Gramineae was verified by blast software. The *x*-axis and *y*-axis represent the mitogenome location (bp) and the order of the transferred sequences, respectively. The black and red lines indicate perfect match regions located in intergenic or partial genic regions. The matched whole genic region was labeled with gene name.

Considering that LCBs analysis is a more suitable tool for identifying large-scale rearrangement, gene gain, and gene loss, BLAST software was applied to check that the fragments throughout the mitogenomes perfectly matched with the databases of their own corresponding chloroplast genomes (Figure 6). The results showed that the percentage of transferred sequences among the nine Gramineae mitogenomes ranged from 2.74% (*E. sibiricus*) to 7.11% (*Sorghum bicolor*). The transferred genes of the mitogenomes almost all belonged to tRNA and rRNA genes, and only the coding gene of *atp4* was transferred between the mitochondrial and chloroplast genome of *Zea perennis*.

### Horizontal Gene Transfer Analysis Across 88 Plant Mitogenomes From the Aspect of Compound Mitogenome Simple Sequence Repeats

**FIGURE 7:**
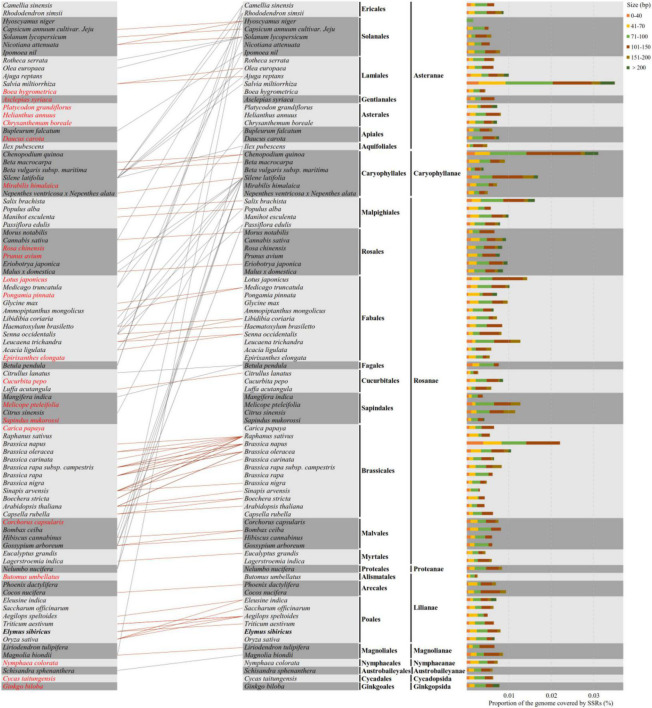
Compound mitogenome simple sequence repeat (SSR) sequences (mt-c-SSRs) identified in 88 mitogenomes of seed plants. The types and proportions of mt-c-SSRs are shown on the right, and the cluster analysis indicating the relationships and mt-c-SSRs transfer among all species is presented on the left. The orange and gray lines show relationships within and between taxa, respectively. Species that have no connection with any other ones are colored in red font.

A total of 88 well-annotated seed plant mitogenomes including *E. sibiricus* were employed for the identification of their mitogenome SSR sequences (mt-SSRs). The identified compound mitogenome SSR sequences (mt-c-SSRs) were applied in the construction of interspecies connections because of their conservation and transferability. The identified mt-c-SSRs were divided into six types based on their length, among which the sizes of 71∼100 bp and 101∼150 bp were the most dominant elements in each species (Figure 7). To evaluate the interspecific relationships among all of the above 88 mitogenomes, a cluster analysis based on all identified mt-c-SSRs was made with the “CD-HIT-EST” online pipeline. A line was connected between two species when the similarity among their equal length mt-c-SSRs achieved 100%. As shown in Figure 7, the 88 species were arranged by their phylogenetic classification. The clustering result revealed obvious synteny within systematic taxa, while inter-taxa relationships were also detected. A total of 21 species had no affinity with any other species; however, the phylogenetic relationships among all of the 88 seed plants could also be detected when reducing the sequence similarity to 90% (Supplementary Data). Specially, *Silene latifolia* was connected with 16 other species distant from its own taxon (Figure 7). In addition, *E. sibiricus* was found to have only sequence transfer with *Aegilops speltoides* and *Triticum aestivum*. All of the mt-c-SSRs were mapped back onto their own mitogenomes (Table 2), of which 11 genes (*atp1*, *ccmFN*, *nad4*, *nad5*, *nad9*, *rps1*, *rps2*, *rps3*, *rps10*, *rrn26*, and *rrnL*) were found to contain some of these mt-c-SSRs, especially, the motif “(ATA)_3_agtcaagtcaag (AAT)_3_” occurred in the *nad5* gene of 8 species.

**TABLE 2:** Genes containing interspecies transferred mt-c-SSRs.

*Species*	mt-c-SSR	Length (bp)	Gene
*Populus alba*	(A)_9_gagaactagatacctttctacaagaaagtgcgttgactttaatataatataat(ATG)_3_	71	*atp1*
*Aegilops speltoides*	(A)_8_ggtgcattagcggttaatacgttgtaattagataagttatttggaatatgacggaacgaaagaccaaggaaag(GAA)_3_	90	*ccmFN*
*Triticum aestivum*	(A)_8_ggtgcattagcggttaatacgttgtaattagataagttatttggaatatgacggaacgaaagaccaaggaaag(GAA)_3_	90	*ccmFN*
*Salvia miltiorrhiza*	(A)_9_taggtgacctaacg(GCC)_3_	32	*nad4*
*Acacia ligulata*	(ATA)_3_agtcaagtcaag(AAT)_3_	30	*nad5*
*Ajuga reptans*	(ATA)_3_agtcaagtcaag(AAT)_3_	30	*nad5*
*Libidibia coriaria*	(ATA)_3_agtcaagtcaag(AAT)_3_	30	*nad5*
*Malus x domestica*	(ATA)_3_agtcaagtcaag(AAT)_3_	30	*nad5*
*Salvia miltiorrhiza*	(ATA)_3_agtcaagtcaag(AAT)_3_	30	*nad5*
*Silene latifolia*	(ATA)_3_agtcaagtcaag(AAT)_3_	30	*nad5*
*Gossypium arboretum*	(ATA)_3_agtcaagtcaag(AAT)_3_	30	*nad5*
*Hibiscus cannabinus*	(ATA)_3_agtcaagtcaag(AAT)_3_	30	*nad5*
*Leucaena trichandra*	(T)_9_agagcaagaagcggaactacaagaaagctttctttatctttatggataaccaatccattttaaaatatagttgggagactttac ccaagaaatgggt(A)_8_	114	*nad9*
*Libidibia coriaria*	(T)_9_agagcaagaagcggaactacaagaaagctttctttatctttatggataaccaatccattttaaaatatagttgggaga ctttacccaagaaatgggt(A)_8_	114	*nad9*
*Aegilops speltoides*	(T)_8_agaagagcttttttgaatggaagaaaagtagtgaaacccgcgatggctactgaataacctcctttga(T)_8_ataataaaccctttgacctttttct tcgttcgccaaatcttcttcagttctatccaagctcggttttgtctgaatctttgtg(GAA)_3_	174	*rps1*
*Triticum aestivum*	(T)_8_agaagagcttttttgaatggaagaaaagtagtgaaacccgcgatggctactgaataacctcctttga(T)_8_ataataaaccctttgaccttt ttcttcgttcgccaaatcttcttcagttctatccaagctcggttttgtctgaatctttgtg(GAA)_3_	174	*rps1*
*Aegilops speltoides*	(A)_9_ttccgttcaaga(AAG)_3_	30	*rps2*
*Triticum aestivum*	(A)_9_ttccgttcaaga(AAG)_3_	30	*rps2*
*Aegilops speltoides*	(TCT)_3_tccccaatagac(GAA)_3_	30	*rps2*
*Triticum aestivum*	(TCT)_3_tccccaatagac(GAA)_3_	30	*rps2*
*Brassica rapa*	(AGA)_3_acgaaacgaagtgagaggccggggggcaaggaaaagagtcgagtcgatcaggctcgacgaccgaaagaagcaaaacgaaa tccgggggtggccg(A)_9_	112	*rps3*
*Brassica rapa* subsp. *campestris*	(AGA)_3_acgaaacgaagtgagaggccggggggcaaggaaaagagtcgagtcgatcag gctcgacgaccgaaagaagcaaaacgaaatccgggggtggccg(A)_9_	112	*rps3*
*Rotheca serrate*	(A)_8_ggttattatacaccccaaccccatcccttgg(A)_9_	48	*rps3*
*Bupleurum falcatum*	(CTT)_3_agagaaaggggaaaggggcaacctatcttac(TAA)_3_	49	*rps10*
*Glycine max*	(CTT)_3_agagaaaggggaaaggggcaacctatcttac(TAA)_3_	49	*rps10*
*Acacia ligulata*	(TTA)_3_gtaagataggttgcccctttcccctttctc(TAA)_3_	48	*rps10*
*Capsicum annuum* cultivar. *Jeju*	(TAA)_3_ggtaagctttcaa(GCC)_3_	31	*rrn26*
*Hyoscyamus niger*	(TAA)_3_ggtaagctttcaa(GCC)_3_	31	*rrnL*
*Hyoscyamus niger*	(GGC)_3_ttgaaagcttacc(TTA)_3_	31	*rrnL*
*Magnolia biondii*	(GCA)_3_gctttgtttgcatgacttcgctcgcagtatggaagattggattcggtagttttcattga(TTC)_3_	77	*rrnL*

Considering the cross-species transfer feature of SSR, the Spearman correlation coefficients among 88 mitogenomes were calculated using a dataset consisting of the numbers of mono-, di-, and tri- nucleotide repeats in each species (Supplementary Figure 4A). Overall, 80% of the interspecific pairwise correlation coefficients were larger than 0.8, which indicated a strong correlation among most species considering c-SSRs. However, two ancient species, *Ginkgo biloba* and *Cycas taitungensis*, and four other species (*Schisandra sphenanthera*, *Nymphaea colorata*, *Magnolia biondii*, and *Liriodendron tulipifera*) possessed relatively lower correlation coefficients with respect to other species. The correlation coefficients of *Ginkgo biloba* and *Cycas taitungensis* were equal at 0.8823, while the interspecific correlation values between those two species and other species were all less than 0.6. Furthermore, *Platycodon grandifloras* and *Cucurbita pepo* also possessed lower values of correlation with other mitogenomes. The proportion of SSRs to mitogenomes in 88 seed plants ranged from 0.005 (*Epirixanthes elongata*) to 0.036 (*Melicope pteleifolia*, Supplementary Figure 4B), among which most of the SSR’s proportion was between 0.01 and 0.015. However, no obvious pattern between the ratio of SSRs and phylogeny relationship was found.

**FIGURE 8:**
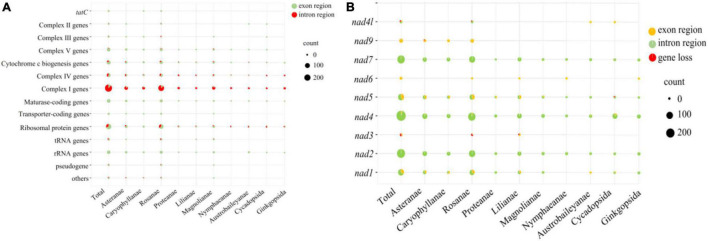
Bubble diagram showing the number of SSRs located in the genic region **(A)** and *nad*- regions **(B)**.

The annotation of the predicted SSRs in 88 seed plants was performed and pooled based on their phylogenetic relationship (Figure 8). The 88 seed plants could be divided into ten superorders. We considered SSRs were located in genic regions, and the vast proportion of SSRs were located in the region of complex I (*nad*-) genes, followed by ribosomal protein genes (*rps*/*rpl*). No SSRs were detected in the intron regions of *tatC*, transporter-coding gene (*mttB*), and rRNA genes across all of the 88 mitogenomes. With the exception of complex I genes, the number of SSRs located in exon regions was larger than those in intron regions. For each of the 10 superorders, the above-mentioned pattern could also be established, and the number of SSRs located in the intron region of complex I genes was about 10 times that of the exon region. Therefore, we further analyzed the distribution pattern of SSRs in complex I genes, including *nad1*, *nad2*, *nad3*, *nad4*, *nad5*, *nad6*, *nad7*, *nad9*, and *nad4l*. Overall, *nad2*, *nad4*, and *nad7* contained the most SSRs, and vast of them were located in the intron region. *Nad3* (3) and *nad6* (4) contained the minimum number of SSRs, and the SSRs they contained were all distributed in the genic region. With the exception of *nad2* and *nad6*, the remaining seven *nad*- genes were lost in some species, of which *nad1* and *nad3* were lost in four species. Especially, *nad1* and *nad3* were both lost in the mitogenome of *Saccharum officinarum*. In contrast to *nad2*, *nad4*, *nad5*, and *nad7*, the other five *nad*- genes only contained SSRs in certain species.

### Phylogenetic Analysis of *Elymus sibiricus* and the Related *E. nutans* Based on Mitochondrial Simple Sequence Repeats

**FIGURE 9:**
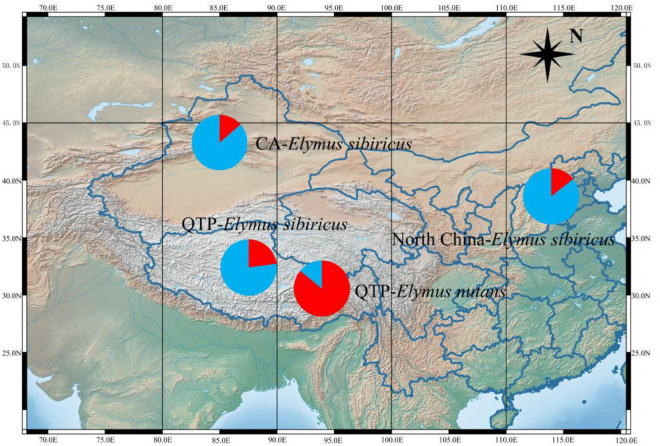
STRUCTURE analysis revealing the genetic affiliations of 60 *E. sibiricus* and 32 *E. nutans* accessions. The red and blue colors indicate the different genetic background.

Fifty mt-SSRs identified from the *E. sibiricus* mitogenome were randomly selected to amplify 60 *E. sibiricus* and 32 *E. nutans* accessions. Five polymorphic mt-SSR markers were further employed to analyze the genetic background of the tested accessions. Following Evanno’s method, the best *K*-value was two in the present study, which indicated that the tested accessions possessed two genetic affiliations (Figure 9). The genetic background of 32 *E. nutans* accessions was obviously different from the 60 *E. sibiricus* accessions, which indicated that the five pairs of mt-SSR markers could be potentially used to distinguish *E. sibiricus* from the morphologically most similar species of *E. nutans*.

### Phylogenetic Analysis of Seed Plant Species Based on Shared Mitochondrial Genes

Considering that no shared mitochondrial gene was found across 88 plant species, we constructed the phylogenetic trees of 79 plant species using their shared mitochondrial genes *atp1*, *ccmB*, and *nad6*, which were all mainly subjected to traditional classification. The Gymnosperm *Ginkgo biloba* and *Cycas taitungensis* were close to the root of all three phylogenetic trees, while it is interesting to note that *Butomus umbellatus* and *Ammopiptanthus mongolicus* were at the root of trees constructed by *atp1* and *nad6*, respectively. To visualize the differences between these phylogenetic trees, tanglegrams were viewed by eGPS software. The results indicated differences in the phylogenetic relationships among certain species, but the relatedness within small clades (systematic taxon) was similar (Supplementary Figures 5–7).

## Discussion

### Mitochondrial Genetic Basis of the Plateau Adaptation of *Elymus sibiricus*

Mitochondrial genomes have been widely utilized in the studies of plateau adaptation in animals (Yuan et al., 2018). Our study complements the knowledge on the adaptive evolution of mitochondrial genomes in high-altitude plants. As one of the dominant species in the alpine meadow ecosystem of the QTP, *E. sibiricus* has been subjected to evolutionary selection pressure for millions of years, such as by high-ultraviolet radiation, low temperature, and growth water deficiency, which led to its excellent stress resistance. However, long-term selective stress can certainly result in the fast accumulation of deleterious or sublethal mutations in mitogenomes because of its uniparental inheritance character, which further cause its doomed shrinkage or death (Saccone et al., 2000). On the other hand, the positive correlation between genome size and extinction rate has been documented in plants (Bennett and Leitch, 2005). The minimum genome size of *E. sibiricus* compared with three other Triticeae species suggests that the mitochondria of *E. sibiricus* may have experienced adaptative shrinkage to better survive the extreme environment of the plateau. Unlike nuclear genomes, the size variation of plant mitochondrial genomes is mainly related to the increase and/or deletion of TE, which was considered as the main driving force of the evolution of genome size (Bennetzen, 2005). However, it seems that the main cause of mitochondrial genome contraction in *E. sibiricus* was not the reduction of TE. Instead, the highest TE content (especially in intergenic regions between *mttB* and *trnP*) of *E. sibiricus* compared with three other Triticeae species may be the reflection of accumulated mutations and structural truncation events, as TE activity can be induced by environmental stresses (Capy et al., 2000).

### Horizontal Gene Transfer Reflected by Compound Mitogenome Simple Sequence Repeats Reveals Distinct Intra-Taxa and Even Inter-Taxa Connections

The simple sequence repeats have long been used as molecular markers for intra-species phylogenetic analysis due to their cross-species transfer characteristic (Rossetto and Henry, 2001), which is akin to HGT. The successful amplification of *E. nutans* based on newly developed SSRs from the mitogenomes of *E. sibiricus* also proved the cross-species transfer characteristic of SSRs. Repeats have been reported to be associated with HGT events in some cases (Sanchez-Puerta et al., 2019). Gandini et al. (2019) recently attempted to find the origination and expansion of short repeat sequences across seed plants, and they suggested that short repeat clusters were shared by closely related species. However, Gandini et al. (2019) focused on short repeats (less than 99 bp), which could miss important information. In this study, mt-c-SSRs were first used to construct the evolutional pattern of 88 seed plant species. We found that the expansion of mt-c-SSRs appears to have taken place in ancestral repeats, since connections were quite frequent in the same taxonomic group (especially in Brassicales and Poales). It is not clear whether the expansion of these shared mt-c-SSRs is the result of HGT or it occurred before HGT. However, it is certain that these mt-c-SSRs lead to a new research direction for HGT, as these species with shared mt-c-SSRs have experienced some degree of gene exchange. In addition, connections between species with distant phylogenetic relationships may be attributed to convergent evolution (Gandini et al., 2019). In our study, a total of 21 species were lacking connection with any other species. However, connections across all of the 88 species were observed when reducing the alignment criterion, which indicated the ancestral origin of mt-c-SSRs and the divergent evolution of seed plant species to adapt to the varied environment (Fey and Laurence Maréchal, 1999). These findings provide a novel perspective that compound SSR could be a potential tool for the study of HGT and interspecies phylogenetic evolution while more plant mitogenomes are needed to reveal a broader pattern.

### The Fast Evolution of Mitochondrial-*nad*-Genes Is Reflected by the Simple Sequence Repeat Dynamics

The simple sequence repeats are believed to play a major role in inducing the genetic variation underlying adaption. Only minor changes including replication slippage and mutation can lead to gene function variation, when SSRs are located in their protein coding regions (Li et al., 2004). Otherwise, the SSRs located in intron regions were also proved to act in the role of *cis*-regulatory elements *via* affecting transcription and mRNA splicing and finally change the phenotype (Ranum and Day, 2002). In this case, selection acting on a gene incorporating repeats would exert a greater influence on repetitive DNA evolution. In this study, the estimation of SSR dynamics within 88 mitogenomes indicated that most mtSSRs were enriched within complex I (*nad*-) genes, especially the intron regions of *nad2*, *nad4*, and *nad7*. This suggests that the three genes were under strong evolutionary selection pressure among the 88 species. The intron regions of *nad7* were used for phylogenetic systematics in *Rhodiola rosea* (Deng et al., 2007) and *Korean ginseng* (Wang et al., 2009), which further indicated its special evolutionary position. As a prevalent physiological phenomenon in higher plants, cytoplasmic male sterility (CMS) was closely associated with the structure of *nad7* and *nad4* genes. Studies have shown that the deletion of the last two exons of *nad7* gene encoding the mitochondrial respiratory chain complex I subunit leads to CMS in tobacco (Ling et al., 2011). Furthermore, the RNA editing imperfection in *nad4* of rice was connected with the abnormalities of pollen grain (Kim et al., 2010). The structural variation caused by abundant SSR in these two genes may be one of the reasons for the formation of CMS. On the other hand, the observed variations in mitochondrial introns of angiosperms in the two introns of *nad4* and an intron of *nad7* (Pruchner et al., 2002) may be attributed to the abundant SSR content, as SSRs are the main sources of genetic variation (Kashi and King, 2006). The connection between SSR and stress tolerance in plants have been reported (Kashi and King, 2006). Nad-proteins are the subunits of the respiratory chain nicotinamide adenine dinucleotide (NADH) dehydrogenase on the mitochondrial membrane, which restricts mitochondrial reactive oxygen species (ROS) production by maintaining the relative oxidation state of electron transport chain (ETC), thus alleviating the damage caused by various stresses to plants (Luo and Song, 2004). The improved salt tolerance in *nad3* overexpressed *Gossypium hirsutum* (Zhang, 2015), and the reduced photosynthesis (Sabar and Kouchkovsky, 2000) and nitrogen metabolism (Dutilleul, 2005) in *nad7* mutant tobacco also proved their crucial roles in stress tolerance. The abundant SSR contents of nad- gene in seed plants may increase their tolerance to stresses, as more repeats at the DNA level might be the driving force of faster adaptation (Trifonov, 2004; Li et al., 2010). However, considering that intronic SSRs can affect gene expression and mRNA splicing (Li et al., 2004), the availability of transcription information will likely help to elucidate the mechanism of SSRs improving the stress resistance.

## Data Availability Statement

The datasets presented in this study can be found in online repositories. The names of the repository/repositories and accession number(s) can be found below: https://db.cngb.org/about/, CNX0375565.

## Author Contributions

YLX and QY: conceptualized the basic idea and plan the study. YX, XL, and WL: helped in data collection and analyses. LL, JMZ, and JBZ: performed the statistical analyses. YP and DL: helped in primary draft preparation. SB and YLX: supervision. YLX, SB, and XM: writing – reviewing and editing. All authors contributed to manuscript revision, read, and approved the submitted version.

## Conflict of Interest

The authors declare that the research was conducted in the absence of any commercial or financial relationships that could be construed as a potential conflict of interest.

## Publisher’s Note

All claims expressed in this article are solely those of the authors and do not necessarily represent those of their affiliated organizations, or those of the publisher, the editors and the reviewers. Any product that may be evaluated in this article, or claim that may be made by its manufacturer, is not guaranteed or endorsed by the publisher.

## Footnotes

1
https://github.com/OpenGene/fastp

2
https://blast.ncbi.nlm.nih.gov/Blast.cgi

3
https://github.com/Nextomics/NextPolish

4
https://www.ncbi.nlm.nih.gov/orffinder/

5
http://lowelab.ucsc.edu/tRNAscan-SE/

6
https://chlorobox.mpimp-golm.mpg.de/OGDraw.html

7
http://www.prepact.de/prepact-main.php

8
https://www.perl.org/get.html

9
http://weizhong-lab.ucsd.edu/cd-hit/

## Supplementary Material

Supplementary Figure 1 The visualization of coverage depth in mitogenome of *Elymus sibiricus*. The coverage depth was showed with the color of dark blue.

Supplementary Figure 2 Synteny between mitochondrial and chloroplast genomes in Gramineae was analyzed using Mauve software.

Supplementary Figure 3 RNA editing sites prediction of the 10 shared genes in Gramineae mitogenomes. A to J indicated RNA editing sites of *atp1*, *ccmB*, *ccmC*, *ccmFN*, *cox1*, *cox2*, *matR*, *nad6*, *nad7*, and *nad9*. All the figures possessed the same order of species with A.

Supplementary Figure 4 Heatmap showing the correction coefficients among the 88 mitogenomes **(A)** and the proportion of SSR sequences to the whole mitogenome **(B)**. The species order from top to bottom in **(A)** and from left to right in **(B)** is consistent with Figure 7. The species in a red box belongs to an Order. The white color and blue color presented relationship coefficients were equal to 0 and 1.

Supplementary Figure 5 The tanglegrams between *atp1* and *ccmB* viewed by eGPS software.

Supplementary Figure 6 The tanglegrams between *atp1* and *nad6* viewed by eGPS software.

Supplementary Figure 7 The tanglegrams between *ccmB* and *nad6* viewed by eGPS software.

Supplementary Table 1 The pairwise Ka/Ks values among the nine Gramineae species.

Supplementary Table 2 The alignment result between chl- and mito- genomes of the nine Gramineae species using Mauve.

Supplementary Table 3 The result of sequence clustering based on CD-HIT-EST.
