# Supplementary material for: The Complete Mitogenome of Elymus sibiricus and Insights Into Its Evolutionary Pattern Based on Simple Repeat Sequences of Seed Plant Mitogenomes
Source: Front Plant Sci. 2022 Jan 26;12:802321. doi: 10.3389/fpls.2021.802321 (PMC8826237; doi:10.3389/fpls.2021.802321)
Supplement: Supplementary Figure 1 — The visualization of coverage depth in mitogenome of Elymus sibiricus. The coverage depth was showed with the color of dark blue. [file Data_Sheet_1.zip › Fig S2.pdf]

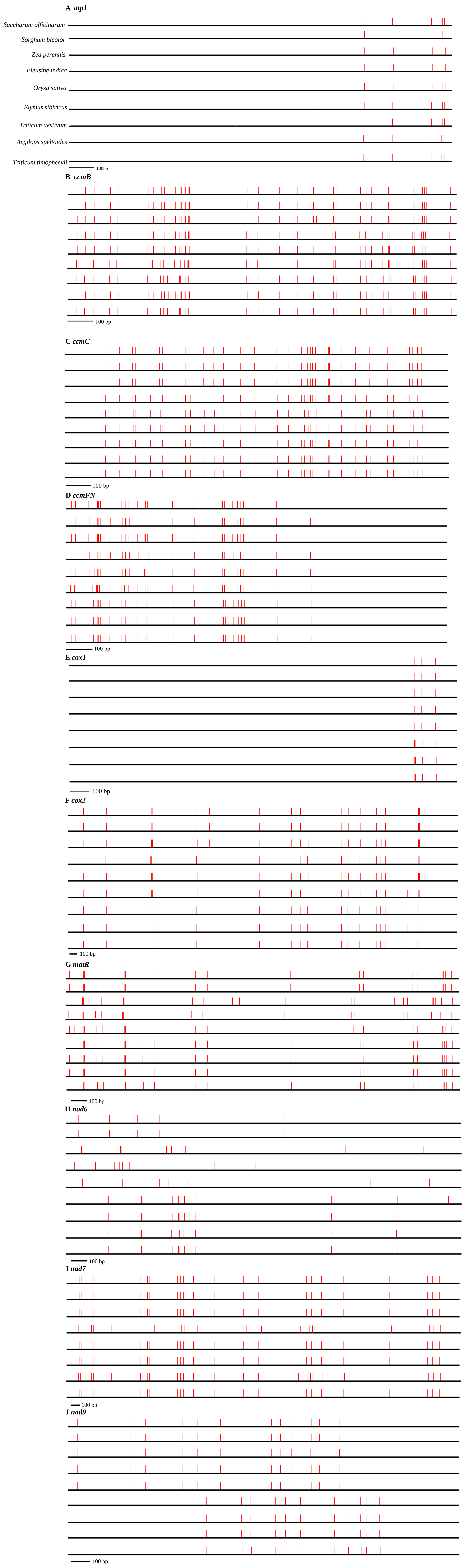

Fig S2 RNA editing sites prediction of the 10 shared genes in Gramineae mitogenomes. A to J indicated RNA editing sites of *atp1*, *ccmB*, *ccmC*, *ccmFN*, *cox1*, *cox2*, *matR*, *nad6*, *nad7* and *nad9*. The horizontal lines in bold black represent the genes in nine mitogenomes, the vertical red lines are the predicted RNA editing sites. All the figures were possessing the same order of species with A.
