# Supplementary figures and images for: The Complete Mitogenome of Elymus sibiricus and Insights Into Its Evolutionary Pattern Based on Simple Repeat Sequences of Seed Plant Mitogenomes
Source: Front Plant Sci. 2022 Jan 26;12:802321. doi: 10.3389/fpls.2021.802321 (PMC8826237; doi:10.3389/fpls.2021.802321)

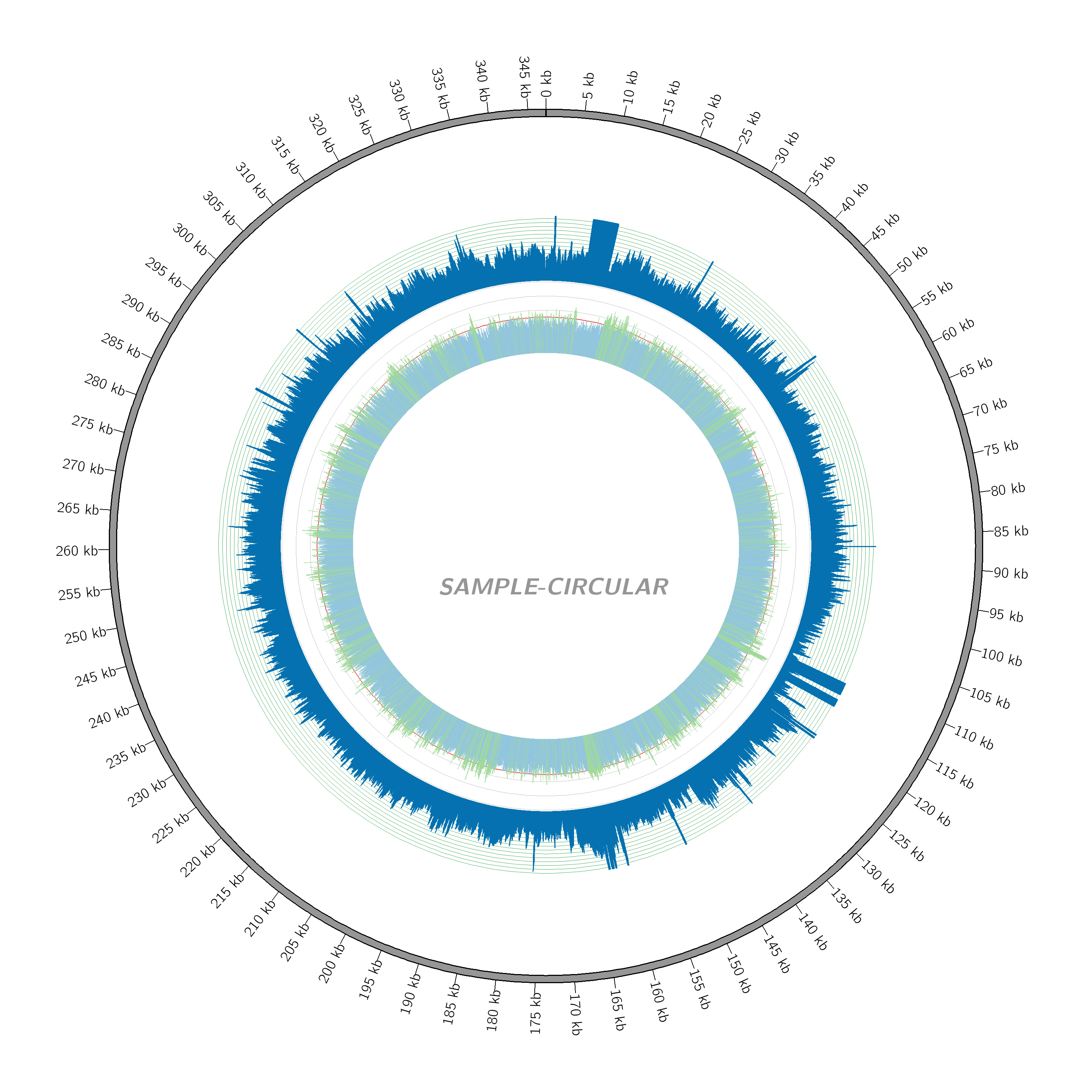

Supplement: Supplementary Figure 1 — The visualization of coverage depth in mitogenome of Elymus sibiricus. The coverage depth was showed with the color of dark blue. [file Data_Sheet_1.zip › Fig S1.png]

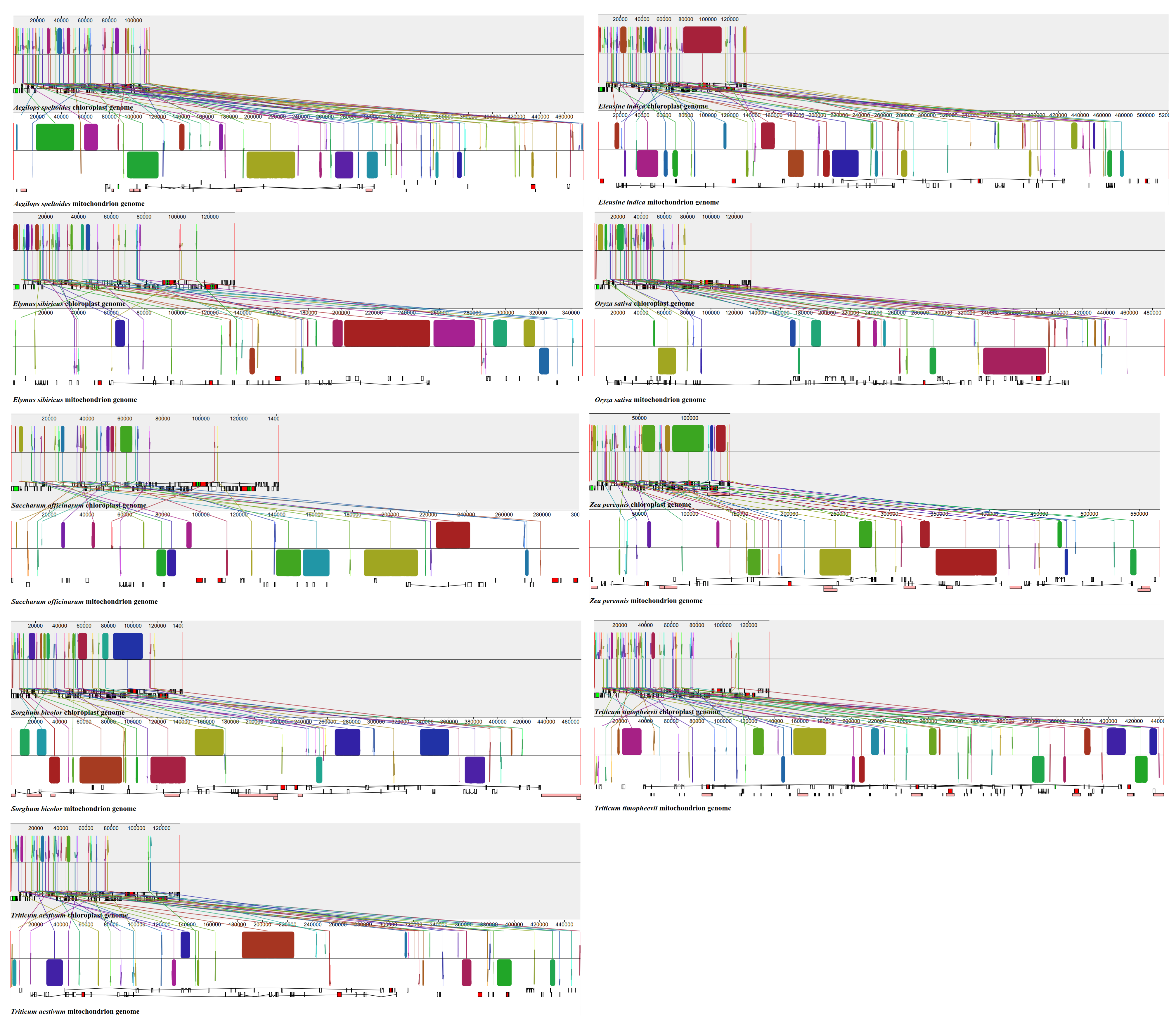

Supplement: Supplementary Figure 1 — The visualization of coverage depth in mitogenome of Elymus sibiricus. The coverage depth was showed with the color of dark blue. [file Data_Sheet_1.zip › Fig S3.tif]

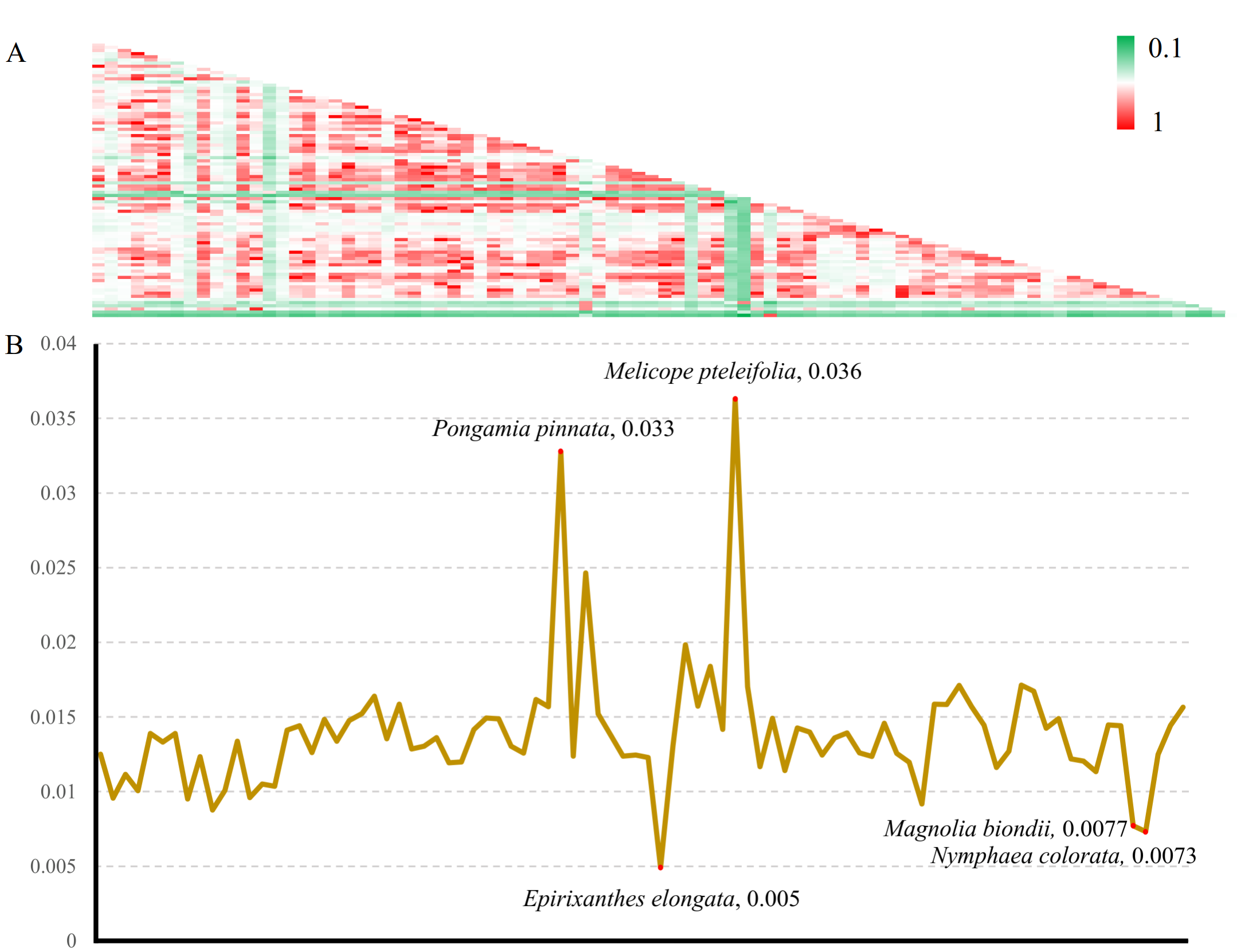

Supplement: Supplementary Figure 1 — The visualization of coverage depth in mitogenome of Elymus sibiricus. The coverage depth was showed with the color of dark blue. [file Data_Sheet_1.zip › Fig S4.tif]

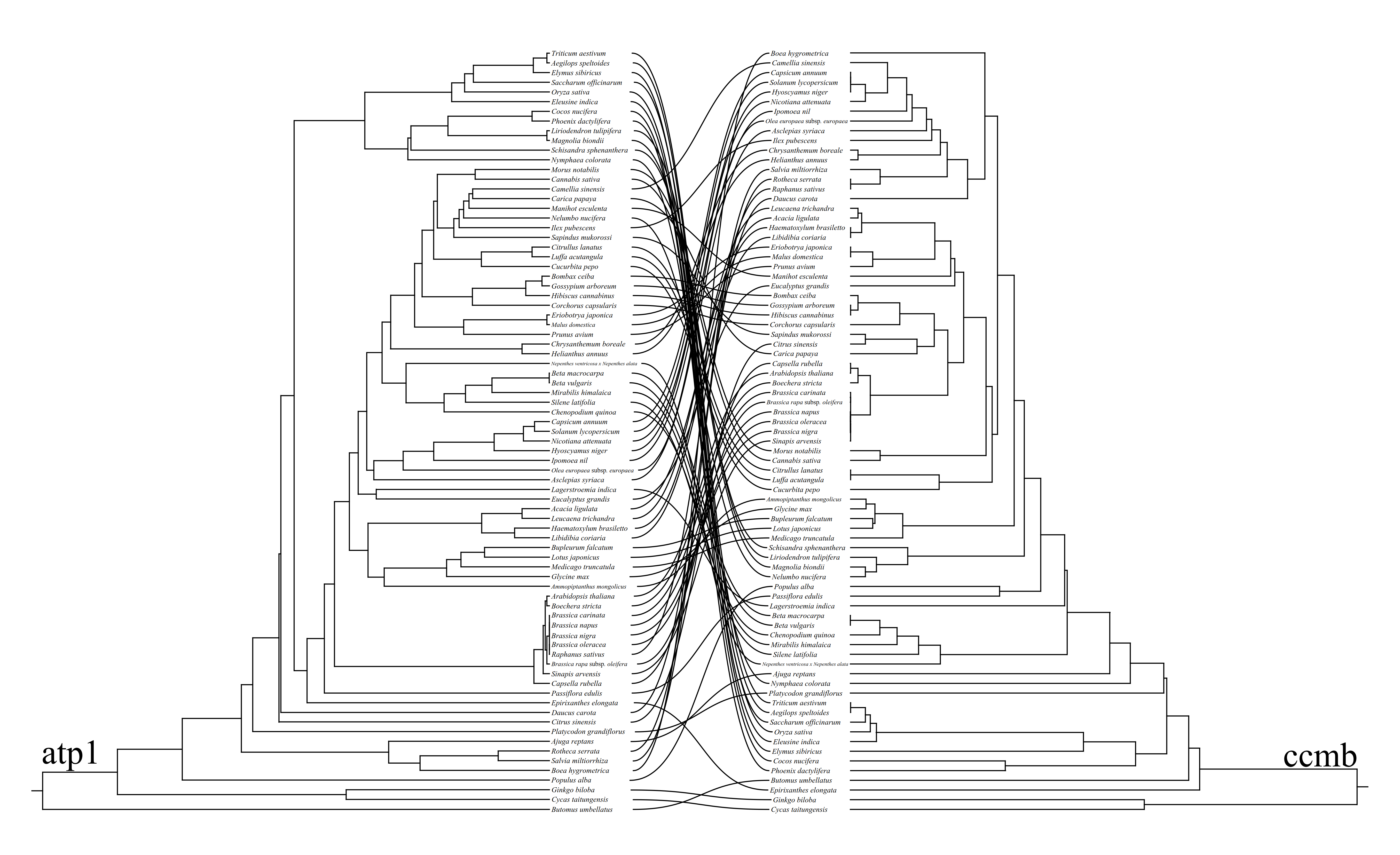

Supplement: Supplementary Figure 1 — The visualization of coverage depth in mitogenome of Elymus sibiricus. The coverage depth was showed with the color of dark blue. [file Data_Sheet_1.zip › Fig S5.tif]

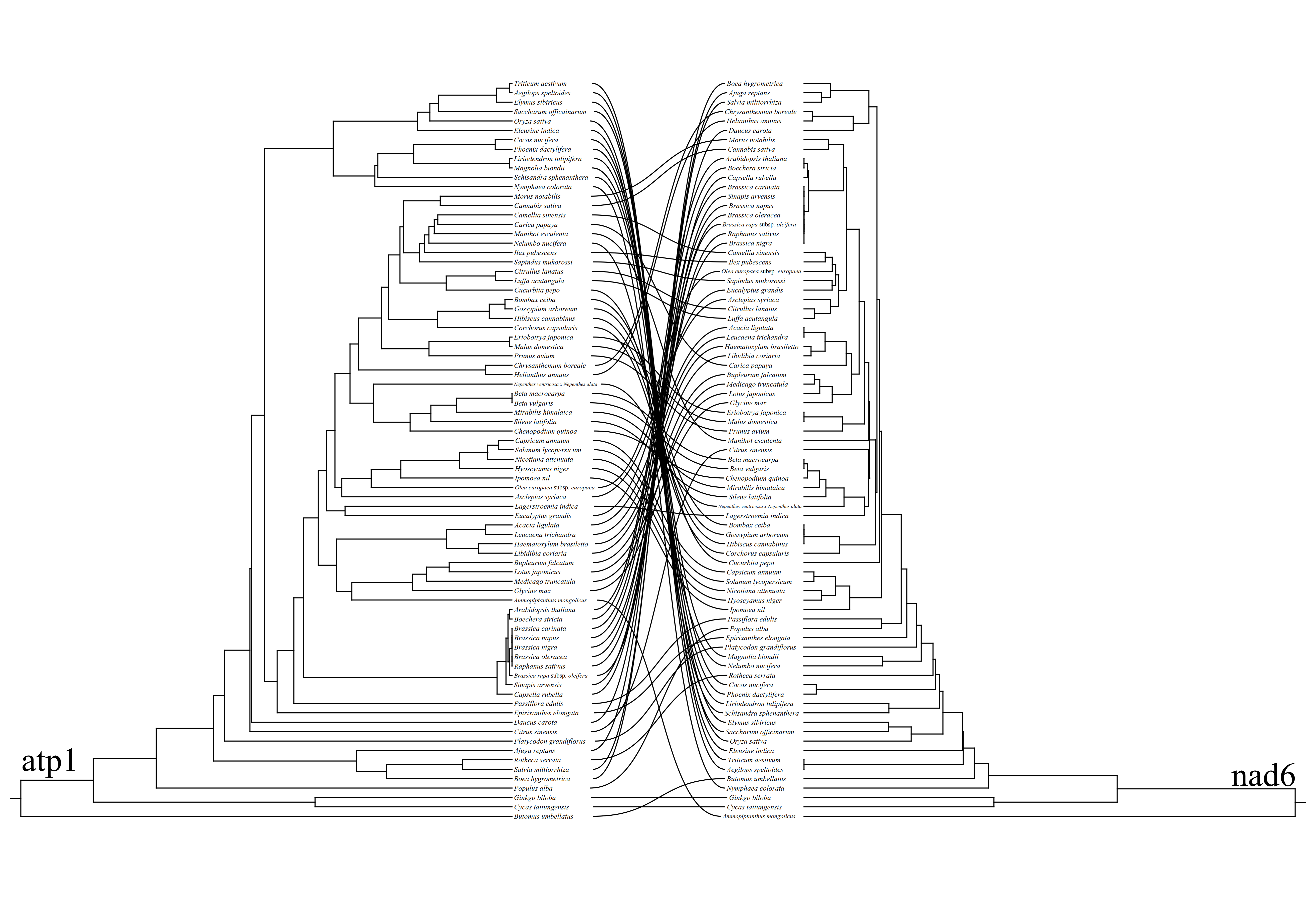

Supplement: Supplementary Figure 1 — The visualization of coverage depth in mitogenome of Elymus sibiricus. The coverage depth was showed with the color of dark blue. [file Data_Sheet_1.zip › Fig S6.tif]

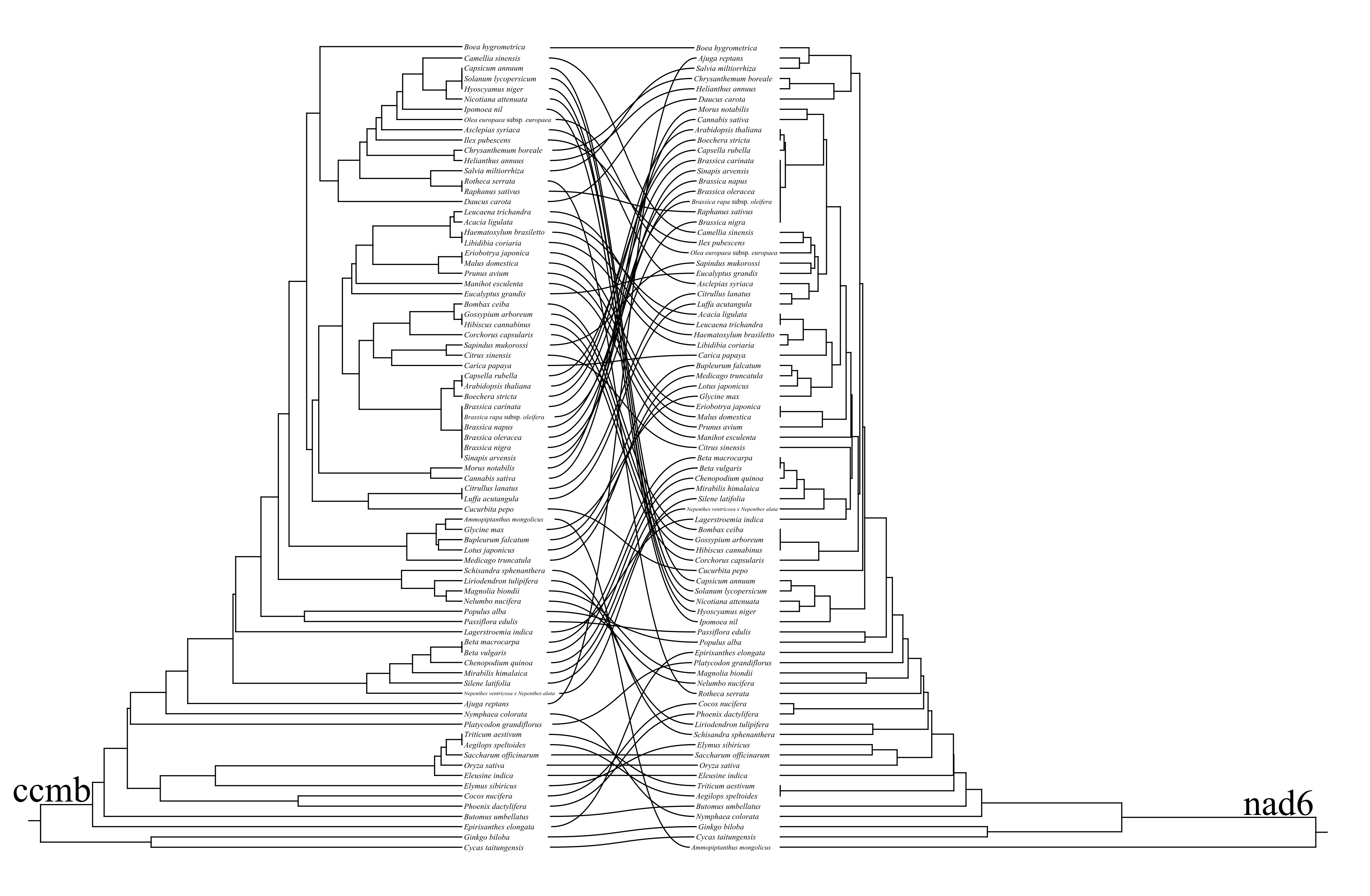

Supplement: Supplementary Figure 1 — The visualization of coverage depth in mitogenome of Elymus sibiricus. The coverage depth was showed with the color of dark blue. [file Data_Sheet_1.zip › Fig S7.tif]
